# Virtual Antenatal Encounter and Standardized Simulation Assessment (VANESSA): Pilot Study

**DOI:** 10.2196/games.9611

**Published:** 2018-05-11

**Authors:** Patrick Motz, Megan Gray, Taylor Sawyer, Jennifer Kett, Douglas Danforth, Kellen Maicher, Rachel Umoren

**Affiliations:** ^1^ Division of Neonatology Department of Pediatrics University of Washington Seattle, WA United States; ^2^ Division of Neonatology Department of Pediatrics Mary Bridge Children's Hospital Tacoma, WA United States; ^3^ Medical Simulation Obstetrics and Gynocology Ohio State University Columbus, OH United States

**Keywords:** prenatal counseling, simulation, ethics

## Abstract

**Background:**

Prenatal counseling at the limits of newborn viability involves sensitive interactions between neonatal providers and families. Empathetic discussions are currently learned through practice in times of high stress. Decision aids may help improve provider communication but have not been universally adopted. Virtual standardized patients are increasingly recognized as a modality for education, but prenatal counseling simulations have not been described. To be valuable as a tool, a virtual patient would need to accurately portray emotions and elicit a realistic response from the provider.

**Objective:**

To determine if neonatal providers can accurately identify a standardized virtual prenatal patient’s emotional states and examine the frequency of empathic responses to statements made by the patient.

**Methods:**

A panel of Neonatologists, Simulation Specialists, and Ethicists developed a dialogue and identified empathic responses. Virtual Antenatal Encounter and Standardized Simulation Assessment (VANESSA), a screen-based simulation of a woman at 23 weeks gestation, was capable of displaying anger, fear, sadness, and happiness through animations. Twenty-four neonatal providers, including a subgroup with an ethics interest, were asked to identify VANESSA’s emotions 28 times, respond to statements, and answer open-ended questions. The emotions were displayed in different formats: without dialogue, with text dialogue, and with audio dialogue. Participants completed a post-encounter survey describing demographics and experience. Data were reported using descriptive statistics. Qualitative data from open ended questions (eg, “What would you do?”) were examined using thematic analysis.

**Results:**

Half of our participants had over 10 years of clinical experience. Most participants reported using medical research (18/23, 78%) and mortality calculators (17/23, 74%). Only the ethics-interested subgroup (10/23, 43%) listed counseling literature (7/10, 70%). Of 672 attempts, participants accurately identified VANESSA’s emotions 77.8% (523/672) of the time, and most (14/23, 61%) reported that they were confident in identifying these emotions. The ethics interest group was more likely to choose empathic responses (*P*=.002). Participants rated VANESSA as easy to use (22/23, 96%) and reported that she had realistic dialogue (15/23, 65%).

**Conclusions:**

This pilot study shows that a prenatal counseling simulation is feasible and can yield useful data on prenatal counseling communication. Our participants showed a high rate of emotion recognition and empathy in their responses.

## Introduction

One out of every ten babies is born prematurely [1]. It has become standard practice for health care providers to offer expectant mothers with premature labor a prenatal consultation. This prenatal consultation addresses the complications of premature birth and gives parents a chance to engage in a dialogue about what to expect for their baby. The prenatal consultation becomes even more critical when babies are very premature and may be born at the limits of medical capacity to successfully provide life-sustaining care, otherwise known as the limits of viability [2]. Families may make life or death decisions based on the information given to them by their health care provider. Prior studies show that most parents wish to participate in decision-making in these kinds of situations [3]. However, parents often cannot recall the therapeutic options that are discussed or find that their decision-making is not impacted by physicians’ predictions of survival and outcomes [3]. Rather, psychosocial influences such as religion, spirituality, and hopefulness more readily guided their decisions [2]. In some cases, up to a quarter of parents prefer to relinquish decision making autonomy, either to physicians or by leaving the situation in “God's hands” [2,4].

Despite the gravity of these conversations, there is evidence that communication during prenatal consultations could be significantly improved, and there have also been calls for a more standardized approach to perinatal counseling [4]. For example, some researchers have proposed a framework with visual aids to help parents better understand the outcomes of babies born at the limit of viability [5]. However, this approach of providing more standardized information does not always meet the needs of parents. Parents need to feel understood and supported as they advocate for their baby in a collaborative and compassionate environment [3].

Over the last two decades, the use of standardized patients (SPs) for health provider communication training has increased [4]. However, their use in prenatal counseling is limited and there is evidence that even with training, SP encounters are prone to recall bias that may lead to inconsistent feedback [6]. Virtual SPs may be a more accessible and cost-effective approach. However, in these emotionally charged conversations, the ability of the virtual patient to project a recognizable emotion is a key element to creating a valid user experience [7]. We hypothesize that a standardized virtual patient simulator called Virtual Antenatal Encounter and Standardized Simulation Assessment (VANESSA), with the capacity to express emotions, will be a feasible approach to developing health care provider communication skills in prenatal counseling.

We set out to achieve the following objectives:

1. Enhance a virtual SP simulator with animations reflecting four primary emotional states.
2. Evaluate the degree to which practicing Neonatologists and neonatal nurse practitioners (NNPs) can correctly identify the virtual SP’s emotional states and the frequency of empathic responses to statements made by the patient.
3. Examine differences in participants’ responses to questions posed by the virtual patient.

## Methods

This observational study was approved by the Seattle Children’s Hospital Institutional Review Board. All attending Neonatologists, Neonatal-Perinatal medicine fellows, and NNPs from the University of Washington and Seattle Children's Hospital were eligible to participate, and all Neonatologists who attended a biweekly neonatal ethics interest group participated. Providers who did not routinely provide perinatal counseling were excluded.

VANESSA, a prototype virtual standardized perinatal patient of a woman pregnant at 23 weeks gestation, was adapted from a medical history-taking virtual patient simulator [8] with animated emotional responses on the Unity 3D platform [9]. The VANESSA simulator was programmed to display the emotions of anger, fear, happiness, and sadness through animations of facial expressions and body language. An SP case and potential responses were developed with input from attending Neonatologists who provided extensive perinatal counseling services (Table 1). Using the VANESSA interface with the potential patient responses programmed, the scenario was deployed to participants [10]. A structured dialogue of the scenario was programmed to include the full potential range of emotions. Potential responses to VANESSA’s dialogue were developed with each set containing both empathic and nonempathic options. Demographic data and feedback on the product were collected using an online survey [11].

**Figure 1:**
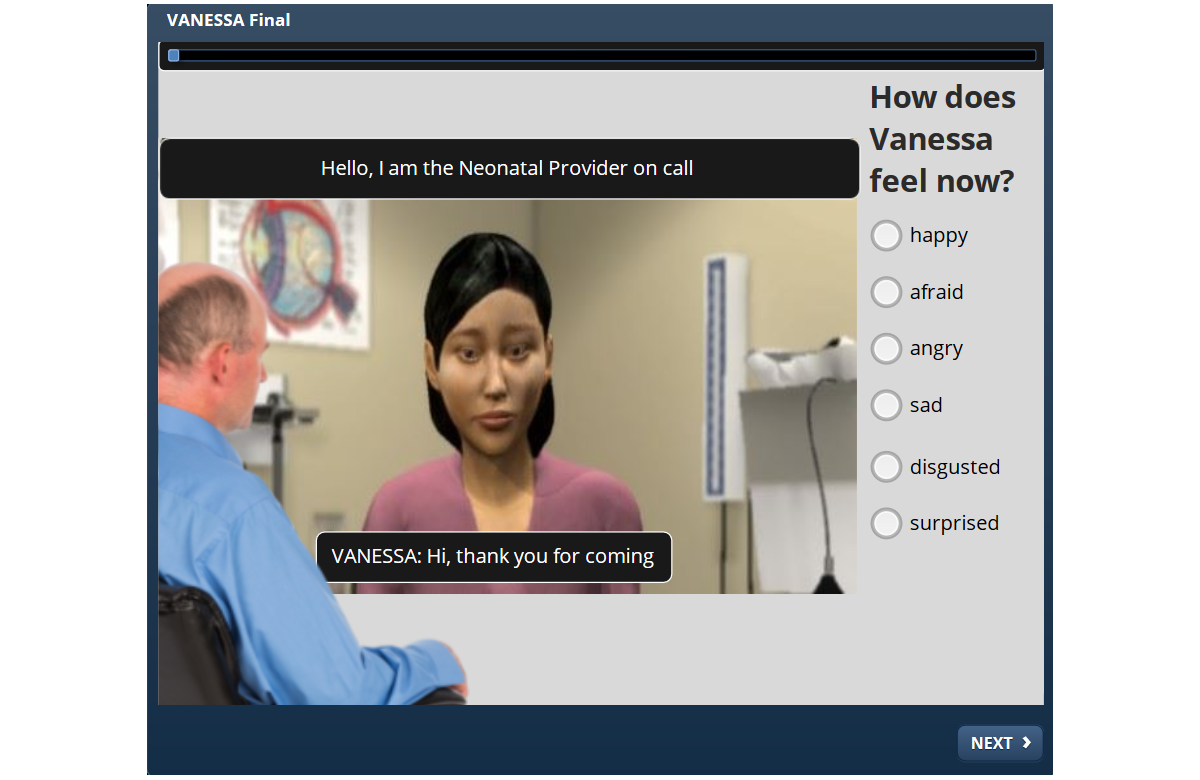
VANESSA prototype interface.

Participants were emailed a link to the online module and associated survey. First, participants were shown video clips of VANESSA displaying emotions with no dialogue (out of context) and asked to identify the emotion she was expressing. Participants were then taken through the prenatal counseling scenario with text displays of both patient and provider dialogue (in context with text) and asked to identify the emotions (Figure 1).

The online module ended with participants participating in the counseling with opportunities to choose responses to the patient’s statements. The interactive counseling section provided audio from the SP and text-based responses to move the scenario forward. Participants were again asked to identify VANESSA’s emotions during the case (in context with audio). The concordance between the displayed emotion and participant responses was determined for each context. At the end of the encounter, participants were asked two open-ended questions by the simulator: (1) “What would you do?” and (2) “What are my options?” Participants responded by typing into a text box. There was no limit to the length of the participant responses.

Once the scenario was finished, the participants filled out a post-encounter survey that included demographic information, years of experience, and formal training in counseling or perinatal counseling. The survey elicited participants’ impressions of the usability of the simulator using a 5-point Likert scale (from 1=strongly agree to 5=strongly disagree).

**Table 1:** Excerpt of VANESSA dialogue.

Topic	Sample Dialogue	Animation
Introduction	Dr. X: Hi, I’m the neonatal provider on-call. Your obstetrician asked me to meet with you to discuss your baby with you. VANESSA: Hi, thank you for coming.	Happy
Assessing patient’s comfort/ interest in talking	Dr. X: Is this an okay time to talk? VANESSA: My husband is gone for the day, but I can talk now. (Patient animation—Sad)	Sad
Assess current understanding	Dr. X: What is your understanding of what might happen in the next few days? VANESSA: It sounds like the baby is coming. I’m only six months along. (Patient animation—Afraid)	Afraid
Relationship building	Dr. X: Have you picked a name for the baby? VANESSA: I’m going to call him Robert, after my dad. Dr. X: That’s a great name. (Patient animation—Happy)	Happy

### Statistical Analyses

Demographic information was analyzed with summary statistics. Multiple choice responses to statements made by VANESSA were collected and analyzed by subgroups including gender, clinical experience, job title, counseling resources used, previous perinatal counseling training, and ethics research focus. Statistical analyses included Kruskal-Wallis and Mann-Whitney tests to evaluate various study subgroups, as the data did not follow a normal distribution. A *P* value <.05 was considered significant. Responses to the open-ended questions were analyzed using a grounded theory analysis approach. Two study team members (PM and RU) evaluated the open-ended responses. An initial list of codes was identified by analyzing the data. Individual codes were discussed further and collapsed into major themes. The final themes were reached after thorough discussion from the two readers. A third study team member (MG) was consulted for discrepancies.

## Results

### Participant Demographics

A total of 24 neonatal providers participated in the pilot study (Table 2). The group was evenly divided between those with less than 10 years of neonatal clinical experience and those with more than 10 years of experience. Most participants reported using medical research and mortality calculators as resources for their perinatal counseling (Table 2). Only the group of Neonatologists who attended a biweekly neonatal ethics interest group listed counseling literature as a resource. Didactic lectures on perinatal counseling and perinatal counseling simulation use were infrequently utilized as resources by participants in this study. Most participants had been previously trained via clinical observation. No participants felt that VANESSA was unnecessarily complex and 96% (22/23) felt they could use VANESSA without the support of a technical person. Few respondents (3/23, 13%) disagreed with the statement that VANESSA was realistic and only one participant felt that she did not respond as other patients would.

### Emotional Identification

**Figure 2:**
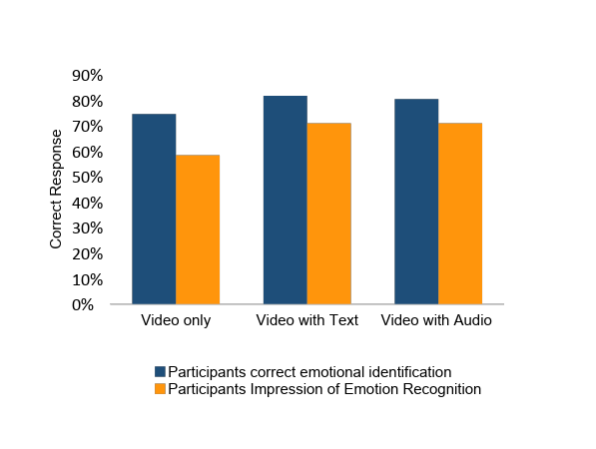
Accuracy of emotion recognition versus participants’ impression of accurate emotional recognition.

**Figure 3:**
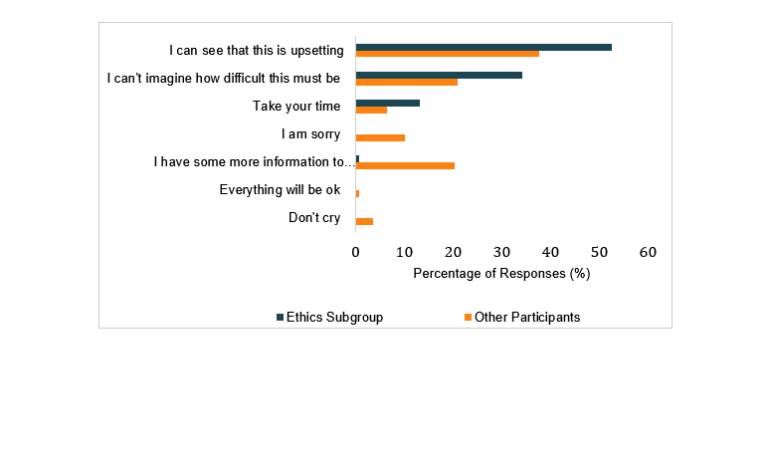
Participants’ empathic versus nonempathic responses.

Of the 672 emotions presented, participants accurately identified VANESSA’s emotion 78.9% (530/672) of the time. As expected, giving participants context through text and audio dialogue did improve their accuracy of emotional identification (Figure 2). When given no contextual dialogue participants were fairly accurate at 74.4% (192/258). By adding text dialogue, respondents improved to 81.7% (291/356) when the context was given. Participants’ confidence in how accurate they were at identifying emotions lagged slightly behind their actual accuracy (Figure 3). When analyzed by each emotion, we found that participants were easily able to identify *happy* (89.8%, 219/244), *afraid* (78.0%, 192/246), and *angry* (80.5%, 161/200) emotions but were less accurate at identifying the *sad* (63.8%, 134/210) emotion.

### Empathic Response to VANESSA

Participants chose empathic responses to VANESSA 75.0% (81/108) of the time. The response chosen most often was, “I can see this is upsetting.” The nonempathic response most often chosen was, “I have more information to share with you, may I go on?” This response accounted for 81% (22/27) of all the nonempathic choices.

The group of Neonatologists who attended a biweekly neonatal ethics interest group were more likely to choose empathic responses (*P*=.01) but were not more likely than the other groups to correctly recognize VANESSA’s emotions. We also assessed differences based on gender, clinical experience, job title, counseling resources used, and counseling training. There were no statistically significant differences between these groups.

### Qualitative Analysis of Participant Responses

The qualitative analysis of the two open-ended questions of, “What would you do?” and, “What are my options?” posed by VANESSA yielded over 50 codes. The codes were distilled into four themes: *eliciting the mother’s values*, *sharing the counselor’s values*, this is a *difficult choice*, and the desire to *give more information* to aid the decision.

**Table 2:** Demographics of study participants.

Demographics		n (%)
**Gender**		
	Male	8 (35)
	Female	16 (65)
**Profession**		
	Neonatal nurse practitioner	7 (29)
	Physician	17 (71)
**Clinical experience**		
	>10 years	12 (50)
	<10 years	12 (50)
**Previous counseling training**		
	Clinical observation	23 (96)
	Workshop	4 (17)
	Simulation	9 (38)
	Communication workshop	6 (25)
**Resources used for perinatal counseling**		
	Medical research	18 (78)
	Counseling literature	7 (30)
	Lectures	5 (22)
	Simulation	3 (13)
	Mortality calculator	17 (74)

Participants focused on *eliciting the mother's values*:

Let's talk more about how you're feeling so I can help you to [the] best answer for your family.

There is no right or “expert” answer, but I am here to help you consider what is best for your baby given your values and unique situation.

*Sharing the counselor's values* was demonstrated through statements such as:

Like you, I would want the best for my baby; whatever that might be.

I would want to make a decision together with my partner. Is there any way for your husband to come be with you?

Participants also acknowledged the *difficult nature of the decision* and recognized that there were different approaches:

This decision is difficult and overwhelming. Everyone approaches these life challenges differently with different priorities.

Few participants gave the definite answer not to resuscitate the soon-to-be-born baby when asked, “What would you do?” Some participants expressed that they didn’t know what they would do if they were in that situation:

I don't know. I've seen very loving families do different things.

Many participants offered more information:

I want to give you as much information as I can, so you and your family can make the decision that is the best for you.

Others reflected the question back to VANESSA in an attempt to elicit her goals and values:

Tell me what is important for your baby and your family.

## Discussion

### Principal Findings

Our study findings demonstrated the feasibility and potential utility of an emotionally expressive virtual perinatal counseling simulator. The “happy,” “afraid,” and “angry” emotions were identified with an accuracy of 80% to 90% (192/246 to 219/244). A high level of emotion recognition by participants interacting with a female virtual SP in a simulated prenatal counseling session is encouraging and is consistent with previous studies that show that recognition of emotion is most accurate on female faces [7,12]. The “sad” emotion was identified accurately only 63.8% (134/210) of the time. This discrepancy persisted despite providing additional text or audio context through scripted conversations. It is possible that the intensity of the “sad” emotion animation was not adequate or that it may have been perceived by participants as a blend of emotions when only one forced choice response was available [13]. Previous studies noted similar accuracy levels for *happiness*, *afraid*, *sadness*, and *angry* using photographic images of facial expressions of emotion [14].

It is important for health care providers to accurately perceive emotions to provide the appropriate support and empathy for patients who are struggling with a diagnosis or those who are coping with a loss. The responses of a subgroup of participants who attended a biweekly neonatal ethics interest group were significantly more empathic toward VANESSA. This finding is consistent with counseling literature that shows that health care providers with interest in ethics had more empathy toward their patients and demonstrates that counseling approaches employed with virtual SPs may parallel those of actual encounters [4].

Virtual prenatal counseling training may be valuable to medical and advanced practice provider training programs. A survey of neonatology program directors revealed an interest in standardizing prenatal counseling training [6]. A prenatal counseling simulator could be used for just-in-time training for residents and fellows and could serve as a way for experienced health care providers to get feedback on their prenatal counseling.

Our qualitative analysis of participant responses to VANESSA's open-ended questions yielded several themes related to the health care providers’ approaches when faced with a difficult question. Most of our participants did attempt to elicit the mother's values rather than presenting their own, but fewer acknowledged that this was a difficult decision with uncertainty in the outcome. Review of the literature notes that families find these two themes to be very important in their counseling [15]. We think this issue underlines the need for further improvement in how we communicate with our patients and underscores the value of virtual SP simulators in research on prenatal counseling, which will be a focus in the next phase of VANESSA's development.

### Limitations

The limitations of our study were that it was conducted at a single academic center and it had a small sample size. Some strengths of our study are that our participants were representative of a large academic neonatology practice, our participants were evenly split between highly experienced providers and moderately experienced providers, and we had participation from both NNPs and physicians.

### Conclusions

In conclusion, this pilot study shows that a perinatal counseling simulation is feasible and can yield useful data on perinatal counseling communication. Our participants showed a high rate of emotion recognition and empathy in their responses. Further work needs to be done to develop our prototype further but demonstrating the recognition of VANESSA’s emotions has laid a solid foundation for additional research to validate this approach.

## Abbreviations

NNP: neonatal nurse practitioner
SP: standardized patient
VANESSA: Virtual Antenatal Encounter and Standardized Simulation Assessment
